# Novel Insights into miRNA in Lung and Heart Inflammatory Diseases

**DOI:** 10.1155/2014/259131

**Published:** 2014-05-27

**Authors:** Amit Kishore, Jana Borucka, Jana Petrkova, Martin Petrek

**Affiliations:** ^1^Laboratory of Immunogenomics and Immunoproteomics, Department of Pathological Physiology, Faculty of Medicine and Dentistry, Palacky University, Hnevotinska 3, 77515 Olomouc, Czech Republic; ^2^Department of Internal Medicine I-Cardiology, Faculty of Medicine and Dentistry, Palacky University, 77520 Olomouc, Czech Republic

## Abstract

MicroRNAs (miRNAs) are noncoding regulatory sequences that govern posttranscriptional inhibition of genes through binding mainly at regulatory regions. The regulatory mechanism of miRNAs are influenced by complex crosstalk among single nucleotide polymorphisms (SNPs) within miRNA seed region and epigenetic modifications. Circulating miRNAs exhibit potential characteristics as stable biomarker. Functionally, miRNAs are involved in basic regulatory mechanisms of cells including inflammation. Thus, miRNA dysregulation, resulting in aberrant expression of a gene, is suggested to play an important role in disease susceptibility. This review focuses on the role of miRNA as diagnostic marker in pathogenesis of lung inflammatory diseases and in cardiac remodelling events during inflammation. From recent reports, In this context, the information about the models in which miRNAs expression were investigated including types of biological samples, as well as on the methods for miRNA validation and prediction/definition of their gene targets are emphasized in the review. Besides disease pathogenesis, promising role of miRNAs in early disease diagnosis and prognostication is also discussed. However, some miRNAs are also indicated with protective role. Thus, identifications and usage of such potential miRNAs as well as disruption of disease susceptible miRNAs using antagonists, antagomirs, are imperative and may provide a novel therapeutic approach towards combating the disease progression.

## 1. Introduction

The small microRNAs (miRNAs), 19–24 nucleotides, are noncoding, endogenous, single stranded, and evolutionarily conserved sequences. miRNAs downregulate gene expression at transcriptional or posttranscriptional level by binding to messenger RNAs (mRNAs) and preventing them from being translated into proteins [1]. They have an important role in biological processes, such as cellular development, differentiation, proliferation, and apoptosis. With the discovery of first miRNA lin-4 in 1993 [2] and later let-7 in 2000 within* Caenorhabditis elegans* during its developmental stage transitions [3, 4], as research advances, the information on miRNAs has grown exponentially and suggested them as one of the central players of gene expression regulation. For nomenclature purpose, they are numbered as per the order of their discovery. As each miRNA is able to target hundreds of transcripts, it has been estimated that miRNAs may regulate up to 30% of the protein-coding genes accounting for 1–5% of all predicted human genes [5]. For humans, to date, over 1,872 precursors hairpin sequences and 2,578 mature miRNAs have been reported (Sanger miRBase version 20; assembly count-GRCh37.p5) which can regulate important physiological processes and pathogenesis of several diseases. This review aims to summarize the current understanding of miRNAs in lung diseases and cardiac remodeling events implicated in inflammation and their potential role as biomarkers.

### 1.1. MicroRNA Biogenesis, Mechanism of Action, and Regulation

miRNAs are transcribed by RNA polymerase II as long precursor (up to several hundred nucleotides) originating as RNA sequences with hairpin structure of about 70–100 nucleotides in length that constitutes the primary transcript of the miR/primary miRNAs (pri-miRNAs). These are further processed in the nucleus by microprocessor complex consisting of RNase III enzyme Drosha and double-stranded RNA binding protein, Pasha (also called DiGeorge syndrome Critical Region 8; DGCR8) to precursor-miRNAs (pre-miRNAs) of approximately 65 nucleotides. These pre-miRNAs are then exported to cytoplasm by exportin-5. This exportin-5 mediated transport to the cytoplasm is energy-dependent, which utilizes GTP bound to the Ran protein as cofactor [6].

**Figure 1:**
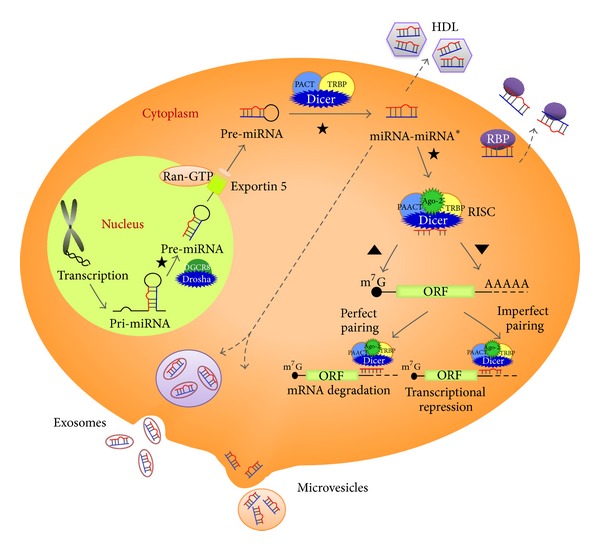
Biogenesis and role of micro-RNA (miRNA). The processing (★) step includes conversion of pri-miRNA to pre-miRNA through Drosha and DGCR8 and pre-miRNA to mature miRNA in the presence of dicer, PACT, and TRBP. In the mature miRNA, either of its strands is involved in RISC formation along with Ago-2. The complex is involved in transcriptional regulation (▲) by binding to site for transcription factors in the 5′-UTR, while it functions (▼) for mRNA degradation (by perfect pairing of its seed region) or transcriptional repression (by imperfect binding) of the target mRNA region. The mature circulatory miRNAs are also transported with microparticles such as membrane derived vesicles (exosomes and microvesicles), lipoproteins (HDL), or RNA binding proteins (RBPs) and remains protected from enzymatic degradation.

In cytoplasm, the hairpin loop is removed and subsequently processed by the RNase III enzyme Dicer along with two double-stranded RNA (dsRNA) binding proteins, protein activator of PKR (PACT) and transactivation response RNA-binding protein (TRBP), leaving mature miRNA duplex (miRNA-miRNA*) of about 22 nucleotides in length with mismatch base [7] (Figure 1). Further, one of the two strands of the mature miRNA is loaded into the RNA-induced silencing complex (RISC) containing enzymes of Argonaute (Ago) family and is commonly guided to the 3′-untranslated region (UTR) of target mRNA. miRNAs anneal with sequences via Watson-Crick base pairing, although there are some examples of miRNA interactions within mRNA coding regions, intron-exon junctions and 5′-UTR. With the activity of Ago-2 where it blocks translation and promotes deadenylation (degradation of poly-A tail) resulting in mRNA degradation, the two mechanisms result in gene silencing [8]. The expression level and involvement of the ribonucleases Drosha, Dicer, and Argonaute-2 (Ago2) in processing and activity of miRNAs demonstrate a relationship with the phenomenon of RNA interference.

Regarding miRNA regulation, in addition to the major stages of control during miRNA biogenesis and its subcellular localization suggested by O'Connell et al. [9] and Yue [10], single nucleotide polymorphisms (SNPs) in the binding domain (seed region) could alter miRNA function. For example, a SNP in the 3′-UTR of asthma susceptibility gene* HLA-G* influences the targeting of miR-148a, miR-148b, and miR-152 [11].

### 1.2. miRNA Polymorphism in Regulation of Target Gene Expression

Mutations in miRNA transcripts are common and SNPs in pre-miRNAs could alter miRNA processing, expression, and/or binding to target mRNA and thus may have functional importance. Numerous reports have been made for the impact of single nucleotide polymorphism in miRNAs (mirSNPs) towards disease susceptibility; however, in this review, focus will be made on inflammatory lung and cardiovascular diseases.

In a case-control study for risk factor for asthma, SNPs demonstrated risk variant rs2910164*C allele (C/G) in the pre-miRNA of miR-146a and rs2292832*T (C/T) miR-149 were significantly associated with lower risk of asthma [12] (Table 1). Corroborating the finding, Jimenez-Morales et al. reported rs2910164*C (G/C) miR-146a to be significantly associated with protection against asthma among Mexican females [13]. However, the rs2910164*C miR-146a was associated with increased risk factor for nasopharyngeal carcinoma [14].* HLA-G* is an asthma-susceptibility gene and within its 3′-UTR rs1063320*G (+13142 C/G) miR-152 family (miR-148a, -148b, and -152) with more stable binding to* HLA-G* mRNA was shown to conserve the miRNA target site and to be protective against asthma only in children of asthmatic mothers. This suggested the allele-specific targeting of HLA-G transcript [11, 15].

Besides, rs11614913*C (C/T) miR-196a2, a prognostic biomarker for shortened survival time [16, 17], was associated with increased risk for several forms of cancers including lung cancer [18–23] and congenital heart disease [24]. Also, rs11614913*T (C/T) miR-196a2 and rs3746444*G (A/G) miR-499 were significantly associated with decreased risk for COPD [25].

In an interesting report from pulmonary tuberculosis (PTB) patients, the roles of risk alleles in miRNAs were reported to vary for disease susceptibility among different populations. Investigating the association of genetic polymorphism with PTB, among the SNPs that regulate the Toll-like receptor- (TLR-) mediated signal pathway, it was shown that rs3746444*C (T/C) miR-499 and rs2910164*G (G/C) miR-146a exhibited different roles in Tibetan and Han populations [26]. Also, miR-146a is* NF-*κ*B*-dependent and regulates cytokine signaling and TLR pathways [27]. Thus, it is highly involved in pathogenesis of several inflammatory and autoimmune diseases and its genetic variants are much studied.

For chronic systemic autoimmune disease, systemic lupus erythematosus (SLE),* SPI1* overexpression is suggested to have a role in its pathogenesis. A SNP rs1057233*T (T/C) in the 3′-UTR of* SPI1* alters a target sequence for miR-569 that is associated with elevated* SPI1* mRNA level and with susceptibility to SLE [28]. However, another variant rs57095329*G (G/A) in the promoter region of miR-146a primary transcript was associated with SLE in Chinese [29]. Understanding the pathogenesis of SLE using combinatorial approach, novel gene-gene/gene-sex interaction was identified which included rs57095329 (G/A) miR-146a. The findings by Leng et al., implicated sex/gender, interferon pathway, and Th17/B cells as important risk contributors to SLE [30]. In a GWAS, rs2431697*T (C/T) was genetically associated with SLE in European population [31]. Further, in a recent study, rs3853839*G (G/C) in 3′-UTR of* TLR7* was reported to affect binding of miR-3148 and was associated with increased risk for SLE [32].

Among the roles of mirSNPs in pathogenesis of cardiovascular diseases (CVDs), the mirSNPs rs5186*C (A/C) in human* angiotensin II type 1 receptor* (*AGTR1*) 3′-UTR affecting miR-155 binding site [35, 36] and rs9818870 (C/T) in* muscle RAS oncogene homolog* (*MRAS*) 3′-UTR modulating miR-195 and miR-135 were proposed to be potentially involved in the hypertension (HT) and other related CVDs [37]. Recently, rs7079 (C/A) in* Angiotensinogen* (*AGT*) 3′-UTR affecting miR-31 and miR-584 were reported to be associated with HT [38].

For coronary heart disease, the GG genotype of miR-149 rs4846049 (G/T) in the 3′-UTR of* 5,10-methylenetetrahydrofolate reductase* (*MTHFR*) was significantly associated with the increased risk [39]. For dilated cardiomyopathy (DCM), rs11614913*T (C/T) miR-196a2 and rs3746444*G (A/G) miR-499 were found to be significantly associated with increased risks [41]. In a genotype-phenotype correlation analysis for congenital heart disease risk, CC genotype of rs11614913 (T/C) miR-196a2 was associated with a significantly increased susceptibility (*p* = 6.81 × 10^−6^) and increased mature miR-196a expression (*P* = 0.001) [24]. Few databases for miRNA-related genetic variants are Patrocles, dbSMR, PolymiRTS, MicroSNiPer, miRdSNP, and dPORE-miRNA [42].

These studies suggest the potentiality of genetic variant in disease susceptibility. In future, the allelic variant with low disease susceptibility could be explored in therapeutic mechanism to manage the disease progression.

### 1.3. miRNA in Epigenetics

miRNAs and antisense RNAs are able to direct epigenetic changes, such as histone modifications (e.g., H3K9me2, H3K9me3, and H3K27me3) and DNA methylation at specific loci, thereby evoking heritable and stable silencing of some mammalian imprinted genes. Histone modification involves Ago1 of RISC and the chromodomain protein Chp1 that recognizes H3K9me [43]. For DNA methylation, methyl groups are transferred to carbon-5 of cytosines by* DNA methyltransferases* (*DNMT1* or* DNMT3A* and* DNMT3B*). Other enzymatic effectors of the epigenetic machinery include histone deacetylases (*HDACs*) and polycomb repressor complexes (*PRC1* or* PRC2*). In general, CpG sites flanking the promoter region are hypomethylated in transcriptionally active genes and hypermethylated in inactive genes.

The downregulation of epigenetically controlled miRNAs as well as epi-miRNAs that target elements of the epigenetic machinery [10, 44] has most notably been reported in cancer cells [45]. The first evidence of epi-miRNAs was reported in lung cancer, where miR-29 (a–c) family was shown to directly target the* de novo* enzymes* DNMT3A* and* DNMT3B* [46]. In this model system, few examples of epigenetic regulation of miRNAs with oncogenic properties include hypomethylation of miR-21 [44, 47] and let-7a-3 [48] and hypermethylation of miR-34b/c [49, 50]. However, hypermethylation of CpG island of miR-34a has been proposed with tumor suppressor role [44, 51].

For the pathogenesis of autoimmune disease, such as systemic lupus erythematosus (SLE), the first report on role of DNA methylation was made during early 1980s with certain medications, such as hydralazine and procainamide inhibiting DNA methyltransferase1 (DNMT1) enzyme activity in CD4+ T cells [52], and was subsequently demonstrated by several studies [53, 54]. A variable expression of regulatory microRNAs in lupus CD4+ T cells due to epigenetics has been described [55]. Overexpression of several miRNAs, such as miR-21, -148a [56], -126 [57], and -29b [58], is reported to affect DNA methylation machinery of lupus CD4+ T cells by targeting* DNMT1* (Table 2). MiR-21 indirectly alters* DNMT1* expression by targeting* Ras guanyl-nucleotide-releasing protein 1* (*RASGRP1*) gene, which mediates the Ras-MAPK pathway upstream of* DNMT1* [56]. However, miR-148a and miR-126 directly inhibit* DNMT1* translation via interaction with its 3′-UTR. Aberrant overexpression of miR-29b [58] and miR-126 [57] in lupus CD4+ T cells causes hypomethylation and overexpression of the methylation-sensitive genes CD11a and CD70, leading to T cell and B cell hyperactivity. Inhibition of such miRNAs expression in CD4+ T cells in patients with lupus caused reverse effects. Thus, the peculiar behavior of miRNAs could be potentially used as a prognostic biomarker for invasive phenotypes of inflammatory diseases.

Despite progress in the area of epigenetic modifications in other pathologies, the role of epigenetic factors affecting miRNA regulation in cardiac inflammatory diseases has still to be investigated. Possible differences among DNA methylation in cardiomyopathic and normal heart have been reported in humans [59] but due to lack of direct evidences the epigenetic regulation of miRNAs still remains elusive [60].

## 2. Role of miRNA in Fibrosis

**Figure 2:**
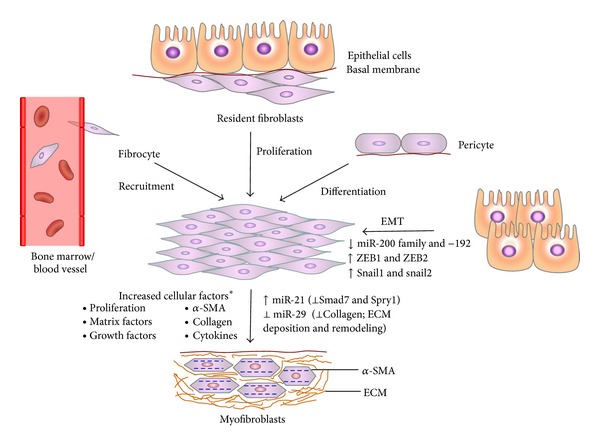
Role of miRNAs in mechanism of fibrosis. The downregulation of miR-200 and -192 (inhibits epithelial-mesenchymal transition, EMT) and miR-29 (prevents the deposition of extracellular matrix, ECM) promotes fibrosis. Further, miR-21 amplifies* TGF-*β** signaling and promotes myofibroblasts phenotype in fibrosis. This is characterized by increased cellular factors* and expression of alpha-smooth muscle actin (*α*-SMA) and EDA-fibronectin.

The hallmark of fibrosis is tissue remodeling with excess deposition of extracellular matrix components, predominantly collagens. Recently, downregulation of miR-200 family (a–c) was reported in the lungs of mice with bleomycin-induced fibrosis; restoration of miR-200 expression reversed lung fibrosis via inhibiting* TGF-*β**, suggesting its antifibrotic role [61]. Upregulation of another miRNA, miR-21, has been related to bleomycin-induced fibrosis (Figure 2). In this case, even the delayed administration of antisense nucleotides blocking miR-21 was able to attenuate the profibrotic effect exerted by bleomycin [62–64]. Vettori et al. reviewed several studies and suggested putative miRNAs as implicated in fibrosis [65]. Amongst these, miRNAs with antifibrotic role in lungs include let-7d and miR-15b, -16, -26a/b, and -29; for heart they include miR-132, -133, and -590; however miR-17~92 cluster (miR-18a, 19a/b), -29a/b/c, and -30c are shared by both lung and heart. miRNAs with profibrotic role in lungs include miR-155, -199a/b, and -23a (clustered with -27a), in heart miR-208; miR-21 appeared in both lung and heart [65]. It has been speculated that targeting of deregulated miRNAs that are implicated in development of IPF, cardiac hypertrophy, and fibrosis may combat the progression of fibrosis in lung and heart.

## 3. Recent Developments in Role of miRNAs in Respiratory Inflammation and Cardiovascular Diseases

### 3.1. Role of miRNAs in Lung Inflammation

An inflammation/injury to lung tissue ignites an innate immune response. Immune cells including macrophages, monocytes, and neutrophils migrate into the lungs to protect the damaging cells and activate antimicrobial peptides and T-cell responses. It further activates proinflammatory response involving cytokines and chemokines, such as* TNF-*α**,* IFN-*γ**, CCL5/RANTES, IL-8, and* IL*-2 as well as innate immune response involving pathogen recognition receptors mediated activation of TLRs and their adaptor proteins such as TRIF, MYD88, TIRAP, and TRAM. These inflammatory stimulators provoke aberrant expression of miRNAs in several chronic inflammatory diseases; for example, miR-146a that targets* COX2* gene is highly increased in response to the stimulation of inflammatory cytokines in several cell types from COPD patients [66], whereas it has reduced levels in CD4+ T- and CD8+ T-cells in patients with severe asthma [67]. A panel of miRNAs such as let-7a and miR-21, -155, -133a, -328, -1291, and -1248 has been shown to differentiate between healthy and inflamed lung in asthma. Additionally, differential expression of specific miRNAs (miR-1248 and -1291) characterized unique miRNA signatures among different types of chronic inflammation such as asthma and COPD [68]. Recently, Sessa and Hata in their review have suggested the role of miRNAs as possible diagnostic and prognostic tool in lung development and pulmonary diseases such as asthma, CF, COPD, IPF, and PAH. They also discussed the potential therapeutic targets for miRNAs using different molecular strategies [8]. Focusing on therapeutic application, Fujita et al. exclusively reviewed the RNA interference (RNAi) in lung inflammatory and cancer diseases along with therapeutic drugs under clinical trials, its route of administration, drug delivery agents, and target gene [69]. The status of miRNAs expression mentioned in this review has been reported to be valid using qRT-PCR or reporter gene assays in majority of the studies. These abnormal immune responses in response to environmental stimuli, infections, and aberrant genetic behavior including miRNA expression may lead to pathological processes and development of various pulmonary diseases [70]. In this review, focus will be made primarily on inflammatory pulmonary and cardiovascular diseases.

#### 3.1.1. Asthma

Asthma is a chronic inflammatory lung disease stimulated by aberrant allergen-specific CD4+ T helper-2 (TH2) secreting cytokines, IL-2, -4, -5, -9, and -13 in response to various stimuli, such as allergens, infections, and air pollutants. It is characterized by elevated serum IgE, airway hyperresponsiveness, mucus hypersecretion, and eosinophil accumulation in the lung [73]. In asthma, upregulated miRNAs include let-7b and miR-21, -106a, -126, -145, -146a, -146b, -155, -181a, and -221, while the downregulated are let-7 family and miR-20b, -133a, -146a, -146b, and -28-5p (Table 3).

During the innate host response to allergens, miRNA expression with elemental regulatory signals has been linked to TLR signaling leading to activation of inflammatory pathways [27]. Notably, miR-21 is among the most overexpressed miRNAs in the inflamed lung tissue and in human airway epithelial cells in response to IL-13 treatment. It suppresses TLR-2 signaling in an animal model of asthma [74]. In a mouse model, it was shown that miR-126 expression was regulated by TLR4 or MyD88 deficient pathways and its antagonism suppressed the effector function of lung TH2 cells along with the development of allergic airways disease [75]. Also, for the bronchial smooth muscle cells, the reduction of miR-133a seems to increase bronchial hyperactivity in an animal model of asthma by increasing the expression of RhoA [76] (Table 3). Further, aberrant expression of miRNAs from primary bronchial epithelial cells including miR-152 family (miR-148a, -148b, and -152) that regulates plasma soluble human leukocyte antigen-G (sHLA-G) is associated with asthma [11, 15]. Recently, substantial differences were demonstrated in exosomal miRNAs profile including let-7 (a–e) and miR-200 (-200b and -141) families between healthy subjects and patients with unprovoked, mild, and stable asthma [77].

#### 3.1.2. Chronic Obstructive Pulmonary Disorder (COPD)

Chronic obstructive pulmonary disease (COPD) is characterized by both chronic inflammation in the airway and systemic inflammation. It is due to combination of emphysema and chronic asthmatic bronchitis leading to impairment of lung function. However, the molecular mechanism of COPD has not been fully elucidated [78]. Proper diagnosis at early stages has remained a major challenge for COPD management. In this context, role of miRNA in COPD development and progression has been well illustrated in several studies (Table 3).

In COPD, miRNAs such as miR-146a and miR-155 have been demonstrated with a regulatory role in inflammation. Cytokine-stimulated prostaglandin E_2_ production and miR-146a expression in cultured fibroblasts correlated with clinical severity of COPD suggesting a pathogenic role of miR-146a [66]. A recent study evaluated the expression of 863 human miRNAs in blood cells of lung cancer and COPD patients along with healthy controls and identified 14 miRNAs as significant for comparing lung cancer and COPD patients [79]. Amongst these, eight miRNAs (hsa-miR-26a, -641, -383, -940, -662, -92a, -369-5p, and -636) were significant for differentiating COPD patients and healthy controls. The differentially expressed hsa-miR-26a acts as regulator of NF-*κ*B pathway by the regulation of its target gene, activating signal cointegrator 1 complex subunit 3 (ASCC3) [79]. Sanfiorenzo et al. identified a six-plasma miRNA panel that was able to discriminate between NSCLC patients and COPD patients and an eleven-plasma miRNA panel that could distinguish non-small-cell lung carcinoma (NSCLC) patients from healthy subjects [80].

#### 3.1.3. Cystic Fibrosis (CF)

Cystic fibrosis (CF) is a monogenic disease caused by mutations in the* CFTR* gene and is characterized by mucus airway obstruction, neutrophil-dominated airway inflammation, and bacterial infection that lead to massive proinflammatory phenotype in the lung. The developmental processes, characterized by bronchial wall thickening and tissues fibrosis, are mediated by the production of reactive oxygen species and metalloproteases [8].

Role of miRNAs in CF has been reported by several workers; among them, Oglesby et al. firstly described miR-126 in CF. In particular, miR-126 targets TOM1 protein, a negative regulator of* IL-1*β**,* TNF-*α**, and* LPS* signaling pathway. The downregulation of miR-126 in CF patients correlated with upregulation of* TOM1* and downregulation of *NF*-*κB*-regulated IL-8 secretion [102]. Moreover, in human airway epithelial cells, three miRNAs, miR-384, -494, and -1246, were shown to inhibit* CFTR* and 3′-UTR of Na^+^-K^+^-Cl^−^ cotransporter* SLC12A2* which is important in regulating chloride transport [100]. Similarly, miR-101 and miR-494 were able to suppress* CFTR* activity by up to 80%, under* in vitro* study [101]. A higher level of miR-155 in CF through hyperexpression of cytokines, such as IL-8 in CF lung epithelial cells in* in vitro* [107, 108] and* in vivo* models [106], suggests the role for miRNA in CF pathogenesis. Further, to understand the aberrant expression mechanism of miR-155 in CF, two mRNA-destabilizing inflammatory RNA-binding proteins, KSRP and TTP, were shown to have an antagonistic role in miR-155 biogenesis [109]. Regarding the biosynthesis of CFTR, miR-138 was shown to alter the expression of several encoding genes associated with CFTR and it also regulates CFTR expression through its interactions with the transcriptional regulatory protein SIN3A [103]. Recently, high expression of miR-101, -144, -145, -215, -223, -509-3p, and -494 in CF cells was shown to be as dynamic regulators of CFTR [98, 104, 105, 108, 110].

#### 3.1.4. Idiopathic Pulmonary Fibrosis (IPF)

Idiopathic pulmonary fibrosis (IPF), defined as a specific form of chronic, progressive fibrosing interstitial pneumonia of unknown cause which is associated with the histopathologic and/or radiologic pattern of usual interstitial pneumonia (UIP) [135], has the largest epidemiological impact and the worst prognosis among interstitial lung disease. Approximately 10% of the microRNAs are significantly deregulated in IPF lungs [111].

In IPF, the regulation of epithelial-mesenchymal transition (EMT) through inhibition of let-7 family members by* transforming growth factor *β*1* (*TGF-*β*1*) and a high expression of* HMGA2* in alveolar epithelial cells were demonstrated both* in vitro* and* in vivo* [111]. Related to progression of fibrosis through myofibroblast differentiation, a high expression of miR-21 was shown in the lungs of bleomycin-treated mice as well as IPF patients and its inhibition reduced the severity of fibrosis [62]. In addition, miR-29 was shown to be involved in EMT and target profibrotic genes in human fetal lung fibroblasts [116] and in bleomycin-induced pulmonary fibrosis in mice [117]. Recently, miR-21 and miR-155 expressions were shown as detectable and stable in serum of patients with IPF [64]. Interestingly, miR-200 family members were shown to reverse the fibrogenic activity of pulmonary fibroblasts in IPF [61]. Comparative analysis of miRNA and gene expression microarray data in IPF revealed enrichment of the* TGF*β*1*,* Wnt*,* Sonic Hedgehog*,* p53*, and* VEGF* pathways and complex regulatory networks [116]. In addition, pathway analysis indicated that altered microRNA expression may be associated with* HGF* signaling, cholecystokinin/gastrin-mediated signaling, and* IGF-1* signaling, among others, in fibrotic lung disease [63].

#### 3.1.5. Other Inflammatory Lung Diseases

Despite the critical role of miRNA in inflammatory response, limited studies have focused on its role in inflammation-induced acute lung injury (ALI)/acute respiratory distress syndrome (ARDS) [122]. The roles of miRNAs such as Let-7a and miR-21, -32, -127, -146a, -155, -181b, -466-5p, and -466-3p have been suggested to be involved in this severe pathology (Table 3). However, the knowledge of this condition has remained limited to establish their role as biomarker for early prevention, prognosis, and therapeutics role in possible therapy; the roles of miRNAs mentioned above are also needed to be validated and confirmed by more studies.

#### 3.1.6. Pulmonary Artery Hypertension (PAH)

Pulmonary arterial hypertension (PAH) is a disease of the pulmonary vasculature characteristic by vascular remodeling associated with obliteration of pulmonary arterioles and formation of plexiform lesions composed of hyperproliferative endothelial and vascular smooth-muscle cells [134]. Wei et al. identified a spectrum of downregulated (miR-451 and -1246) and upregulated (miR-23b, -130a, and -191) miRNAs in PAH patients, suggesting these circulating miRNAs as potential biomarker for early disease detection [128]. Besides, several miRNAs (miR17/92 cluster, -21, -23b, -130a, and -145) detected in PAH were reported to be connected with disrupted BMPR2 pathway in PAH (Table 3). Sarkar et al. confirmed the role of miR-21 in smooth muscle cell proliferation and migration with increased expression of miR-21 in pulmonary artery smooth muscle cells (PASMCs) [126]. By contrast, miR-204 expression in PASMCs was downregulated in both human and rodent PAH model, and delivery of the synthetic miR-204 significantly reduced disease severity in animal model [131]. Similarly,* in vivo* administration of miR-21, -424, and -503 ameliorated PAH [134, 136]. Collectively, these observations propose reestablishment of normal miRNA level as a potential therapeutic approach.

### 3.2. Role of miRNAs in Cardiac Remodeling

Inflammation plays a key role in cardiac function and remodeling during progression of cardiovascular diseases (CVDs). Biological processes affecting fibroblasts, extracellular matrix proteins, coronary vasculature, cardiac myocytes, and ionic channels are involved in this remodeling process [137, 138]. The vascular cell adhesion molecule-1 (VCAM-1) expression is induced through NF-*κ*B mediated pathway and proinflammatory cytokines such as IL-1*β*, IL-6, and TNF-*α*. The VCAM-1 as well as chemokine receptor-2 (CCR2) induces the interaction of vascular endothelial cells (ECs) with monocytes and T lymphocytes which triggers the early atherosclerotic plaques [139, 140]. At transcriptional level, understanding of miRNAs biological functions in the cardiovascular system in physiological and pathological condition is considered to be potentially crucial for CVD prevention, diagnosis, and therapy [138, 141]. For miRNA profiling of cardiac remodeling in response to inflammation, serum based extracellular miRNAs have been extensively utilized as noninvasive and reliable diagnostic tool (Table 4). The pathological process of the myocardium is associated with an altered expression profile of genes that are important for cardiac function. Regulation of cardiac gene expression is complex, with individual genes being controlled by multiple enhancers that regulate specific expression patterns in the heart. Increasing evidences indicate that miRNAs play important role in myocardial pathology adding a new point of view of how cardiac gene expression is regulated.

Recent reviews provide an overview of specific miRNA signatures with dysregulated level in CVDs [138]. These are transported as extracellular microRNAs during cell-to-cell RNA communication and considered as important diagnostic markers [179, 193–195]. For example, Small and Olson explored the role of miRNAs in heart development and pathological cardiac remodeling along with their potential therapeutic targets. They also suggested exciting possibilities for the therapeutic manipulation of miRNA-regulated processes in several cellular mediated diseases that are difficult to modulate therapeutically [196]. Further, extending this approach for therapeutic inhibition of cardiovascular miRNAs, van Rooij and Olson summarized the current chemistries (tiny LNA, LNA-DNA miximer, antagomir, and 2′-F-2′-MOE miximer) for targeting miRNAs [197]. In a study, significant increase in serum level of miR-1 and -133 in patients with cardiovascular diseases such as acute myocardial infarction, unstable angina pectoris, and cardiomyopathy indicated its implication in myocardial damage [198]. In particular, specific set of miRNAs is involved in atherosclerosis, myocardial infarction, heart failure, myocardial hypertrophy, and fibrosis. Here, we summarize and highlight some of the most investigated miRNAs in this field (Table 4), focusing on the main groups of miRNAs involved in myocardial remodeling.

#### 3.2.1. Atherosclerosis

Atherosclerosis (AS), a chronic inflammatory disease affecting major arteries, represents one of the causes of myocardial infarction, ischemic stroke, and peripheral artery disease [199]. miRNAs, such as miR-21, -146a, and -155, are involved in most of the inflammatory diseases [142] and have been implicated in the development of AS; for example, miR-21, -34a, -146a, -146b-5p, and -210 were significantly upregulated in vulnerable atherosclerotic plaques [143] (Table 4). While miR-155 was shown to promote AS by repressing* Bcl6* in macrophages, its inhibition reduced the expression of* CCL2*, which recruits monocytes to atherosclerotic plaques [200]. Mechanistically, miR-155 (proinflammatory) and miR-146 (anti-inflammatory) were reported to regulate dendritic cell functions in atherosclerotic inflammation [201]. As far as the functional aspects of this process, miR-155 is upregulated by macrophage-derived miR-342-5p through* Akt-1* inhibition, which results in stimulation of inflammatory mediators such as* Nos2* and* IL6* in macrophages [202]. On adverse, miR-155 was also suggested to possess protective feature as it prevented development of AS and its progression by regulating inflammatory response and* MAP3 K10* of MAPK pathway [203]. This contrasting effect of miR-155 may be context dependent and differ between early and advanced stages [204]. Other miRNAs implicated in AS, for example, miR-27 [201] or miR-214, were shown to possess cardioprotective effects against ischaemic injury in mouse model by controlling excessive calcium influx and cell death [205].

Also, suppression of NF-*κ*B signaling and inhibition of atherosclerotic lesion by systemic delivery of miR-181b in apolipoprotein E-deficient mouse model with reduced expression of miR-181b suggested the protective role for AS [146]. The two different miRNAs from different sources (human or mouse), sharing a common seed sequence essential for target recognition and binding, have been demonstrated to exert similar role. Recently, inhibition of miR-712 by anti-miR-712 was reported to rescue TIMP3 expression and prevented arthrosclerosis in murine model. From comparative analysis, human miR-205 was also found to have the same seed sequence that targets TIMP3 [147]. Further, they suggested that targeting these mechanosensitive “athero-miRs” and systemic delivery of miRNAs may serve as novel therapeutic approach to treat chronic inflammatory diseases such as atherosclerosis.

#### 3.2.2. Acute Myocardial Infarction

Acute myocardial infarction (AMI) is complex diseases that result from interplay between genetic and environmental factors [206]. Cardiac regulatory proteins, cardiac troponins T (cTnI) and I (cTnI), that control the calcium mediated interaction between actin and myosin have been currently the preferred markers for myocardial injury due to their high sensitivity and specificity for the diagnosis of AMI. However, there is still need for early clinical diagnostic markers for AMI. Further, circulating miRNAs are believed to be closely linked to myocardial injury; moreover, due to the cell-specific physiological functions and miRNAs stability in plasma, serum, and urine, they are being explored as sensitive biomarkers of AMI [207]. Ai et al. observed upregulation of muscle-enriched miR-1 and proposed it to be potential predictor of AMI [149]. Additional miRNAs that were found to be upregulated in patients with AMI included miR-133a, -133b, -208b, -499, and -499-5p, whereas miR-122, -223, and -375 were lower than in controls [150, 151, 156].

#### 3.2.3. Myocardial Hypertrophy and Fibrosis

Cardiac hypertrophy is characterized by an increase in cell size and/or myofibrils without change in myocyte number. Among miRNAs involved in myocardial hypertrophy/fibrosis, miR-133 was suggested to play a fundamental role. Its downregulation was associated with myocardial hypertrophy in mouse and humans [167, 168]. A reduced expression of miR-133a was observed in experimental model of angiotensin II-dependent hypertension in both myocardial hypertrophy and fibrosis. In fact, mir-133a targets collagen 1a1 (Col1A1), which represents the main collagen fibres involved in myocardial fibrosis observed in angiotensin II-dependent hypertension [174]. Downregulation of miR-133 could promote fibrosis by targeting connective tissue growth factor, a potent profibrotic molecule. A reduced expression of miR-133b was observed in all patients regardless of heart failure etiology [208] and during cardiac hypertrophy [209].

Concerning circulatory miRNAs, miR-29a is a common marker for both cardiac hypertrophy and fibrosis reported to be upregulated in patients with hypertrophic cardiomyopathy [165]. MiR-29 family was also related to cardiac fibrosis. Kin et al. found upregulated miR-29 in abdominal aortic aneurysm [173] (Table 4), and van Rooij et al. observed dramatically downregulated miR-29 in the region of fibrotic scar after acute myocardial infarction [210]. Another miRNA, miR-30, regulates a key profibrotic protein, connective tissue growth factor (CTGF) which contributes to collagen synthesis. It downregulates CTGF level and thus controls structural changes in the myocardium extracellular matrix [176]. Similarly, another target of miR-30,* beclin-1* (an autophagy-related gene), also exhibited an inverse relationship. The overexpression of* beclin-1* gene promotes Angiotensin II-induced myocardial hypertrophy by downregulation of miR-30 in cardiomyocytes through excessive autophagy [166].

#### 3.2.4. Heart Failure

The pathogenesis and clinical manifestations of heart failure are complex and involve disruption of normal mechanisms that regulate cardiomyocyte gene expression, growth, survival, and function. Cardiac interstitial cells and vascular cell also actively participate in disease process, resulting in altered myocyte-nonmyocyte signalling, cardiac fibrosis, and decreased vascular density. Currently only B-type natriuretic peptide (BNP) and pro-brain natriuretic peptide (NT-proBNP) are clinically established diagnostic biomarkers for heart failure. However, evaluation of HF progression along with the appropriate timing for therapeutic interventions in the HF patient is important from the perspective of clinical management [185] and new markers are therefore needed.

In this context, signature expression patterns of specific miRNAs that are consistently aberrantly expressed in heart failure patients were described: miR-1, -29, -30, -126, and -133 are found to be downregulated in heart failure patients, whereas miR-21, -23a, -125, -210, -195, -199, and -423-5p are among the upregulated [182–184]. Further, circulating levels of plasma miR-16, -20b, -93, -106b, -223, and -423-5p were significantly increased during hypertension-induced heart failure in mouse model [185]. Plasma concentration of miR-423-5p was significantly elevated in heart failure patients due to dilated cardiomyopathy (DCM) and positively correlated with NT-proBNP level [190]. In a recent study, biopsy of the right atrial myocardium tissue from 83 patients revealed enrichment of miR-1 and miR-133 within cardiac muscle and the decreased level of miR-133a was associated with signs of heart failure [187].

Among several miRNAs, miR-21 was implicated in several cardiac remodelling issues (Table 4). It is expressed in all characteristic cardiovascular cell types, including vascular smooth muscle cells, endothelial cells, cardiomyocytes, and cardiac fibroblasts, and target genes such as programmed cell death 4 (*PDCD4*), phosphatase and tensin homology deleted from chromosome 10 (*PTEN*), sprouty1 (*SPRY1*), and sprouty2 (*SPRY2*) involved in proliferative vascular disease [211]. Reports indicate miR-21 to be involved in pathogenesis of* in vitro* cardiomyocyte hypertrophy and indirectly* in vivo* via fibroblasts. In contrast, some studies report an antihypertrophic effect of miR-21 in isolated cardiomyocytes, a reduction in infarct size by miR-21, or an inhibition of H_2_O_2_-induced apoptosis of isolated cardiomyocytes [212]. Also, miR-21 exhibits a protective role by reducing myocyte apoptosis and ischemic heart failure/reperfusion injury through suppression of phosphatase and tensin homolog (*PTEN*), a negative regulator of the AKT pathway [171, 172]. Thus, owing to its role in both pro- and antihypertrophic, the role of miR-21 in cardiac disease remains controversial. The reasons for the discrepancy between these studies are unclear; however, miR-21 is expressed predominantly in cardiac fibroblasts, not cardiomyocytes [170, 212], primarily due to comparatively increase in the number of cardiac fibroblast cells. Overall, it is indicated that miR-21 inhibits endothelial cell proliferation and migration, whereas it promotes cardiomyocyte and fibroblast survival upon myocardial ischaemia/reperfusion. The potential role of miR-21 in cardiac fibrosis and hypertrophy is still debated and measures such as global knockdown or overexpression of miR-21 in the reperfused myocardium are suggestive to provide an insight on postinfarct remodeling [212].

## 4. miRNA as Biomarker

Aberrant miRNA expression has been associated with various human diseases and its determination can differentiate between normal and diseased tissue [213]. Besides cancer, altered miRNA expression has been reported in lung inflammatory diseases such as asthma, chronic obstructive pulmonary disorder (COPD), cystic fibrosis (CF), idiopathic pulmonary fibrosis (IPF), pulmonary artery hypertension (PAH), and cardiovascular diseases such as atherosclerosis, myocardial infarction (MI), cardiac fibrosis, and coronary artery disease [8, 213]. Banerjee and Luettich reviewed the miRNAs as potential biomarkers for the major smoking-related diseases including cancer, COPD, PAH, and cardiovascular diseases [213]. Also, based on observations from human and mouse model they suggested miRNAs as regulators of biological responses, such as inflammation innate immune response, including TLR signaling. Identification of particular miRNAs within cells, tissues, or cellular free body fluids, with altered expression correlated with disease and/or its clinical development, could thus be exploited as potential biomarker for diagnosis and management. The potential of miRNAs to serve as biomarkers is further supported by their innate characteristics, such as high conservation between species, presence of highly stable cell-free form in the circulation, and omnipresence—being isolated from most of the cells, tissues, and body fluids including serum, plasma, urine, saliva, breast milk, tears, semen, exhaled breath condensate, and bronchoalveolar lavage fluid (BALF); importantly they can be detected in small sample volumes using quantitative real-time PCR (qRT-PCR) [5]. The observed stability, that is, resistance to enzymatic RNase A digestion and other conditions such as boiling, extreme pH, extended storage, and several cycles of freeze-thaw in serum [214] as well in plasma, is also relevant in this context [215].

### 4.1. Circulating miRNAs

Circulating miRNAs, also known as extracellular miRNAs, are cellular free in nature. The origin of circulating miRNAs, stable existence in extracellular environment, and their distinct roles, has remained elusive [193, 216]. They could be divided into two major groups, microparticles associated and microparticles nonassociated miRNAs [217, 218]. Circulating miRNAs are exported from cells and are transported with microparticles such as membrane-derived vesicles (exosomes and microvesicles), lipoproteins (HDL), and other ribonucleoprotein complexes such as* Nucleophosmin-1* (*NPM-1*) and Ago2-miRNA (Figure 1) that protect them against enzymatic degradation [193, 219]. Arroyo et al. characterized circulating miRNA complexes in human plasma and serum. For miRNA profiling, they quantified 88 plasma and 66 serum miRNAs by RT-qPCR and revealed that the vesicle-associated plasma miRNAs represent the minor fraction, whereas up to 90% of circulatory miRNAs were present in a nonmembrane-bound form with ribonucleoprotein complex [217].

Importantly, there are differences in miRNAs expression and abundance between the source, that is, serum and plasma and/or body fluids/other components [220–222]. For example, in the same individuals, higher miRNA concentrations were obtained from serum samples compared to the corresponding plasma samples. This difference was suggested due to release of additional miRNAs from blood cells into serum during the coagulation process. Plasma was suggested as sample of choice, representing true repertoire for circulatory miRNAs [221]. Still, concentration of circulating miRNAs in plasma may be affected by multiple factors such as sample processing, release by specific cells into the circulation, and also miRNA stability [223]. Recently, miRNAs from exosomes [77] and platelets [224] have also been explored and have distinct expression profile [218]. For research, one should consider in which components (body fluid types and microparticles) circulating miRNAs are investigated.

The differential expression of tissue-specific miRNAs in circulation has been explored as potential circulating biomarkers for specific organ pathologies involved in lung or heart disease, for example, skeletal muscle specific miR-1, -499, -133, and -206 in plasma of COPD patients [94] and cardiac specific miR-133a, -208a, and -499 in MI [154, 159, 160, 223]. Most of the miRNAs are significantly detectable in the diseased serum or plasma samples and this thus supports the possibility of using the expression levels of these organ-specific circulatory miRNAs as biomarkers for site-specific pathologies [221].

However, there are limitations and factors to be addressed prior to application of miRNAs in diagnostic purpose. These include optimal standardization in the approaches for obtaining sample (invasive/noninvasive), source (local/systemic), and nature (extra-cellular/cellular/tissue-based) of biological material and minimal variability in sample collection and its processing. Moreover, lack of data on miRNA specificity/sensitivity among different reports and its overlapping role in different diseases also hampers its applicability.

### 4.2. Current Developments in miRNA Based Approaches

The preliminary step includes quality estimation of miRNA. Among the few possibilities, 2100 Bioanalyzer, a lab on-chip technology from Agilent Technologies, offers both qualitative and quantitative estimation of miRNAs. For miRNA discovery, high-throughput deep sequencing (next-generation sequencing) platforms such as HiSeq 2000/Genome Analyzer IIX/Solexa (Illumina), SOLiD (ABI), GS FLX+, or 454 sequencing (Roche) are available [225]. Amongst these, HiSeq 2000 solid phase technology supports massively parallel sequencing using a reversible terminator based method with least error rate and is most successful and widely adopted [226]. In the process of identification of miRNAs, several computational approaches have been developed that complement the experimental analysis. These* in silico* resources include MiRscam, miRSeeker, Phylogenetic shadowing, miRank, miRDeep, MiRanalyzer, proMiR II, mir-abela, triplet-SVM, Vmir, RNA micro, BayesMiRNAFind, One-ClassMirnaFind, miPred, Srnaloop, and findMiRNA [226]. Additionally, miRandola, an extracellular circulating miRNA database, allows users to deduce their potential biological functions and their relation with phenotypes [227].

Most commonly used techniques for establishing miRNA signatures in body fluids include high throughput determination of differential miRNA gene expression using miRNA microarrays [228] and their further validation using quantitative real-time PCR (qRT-PCR). However, the best approach for absolute quantification is qRT-PCR and its two variations, that is, stem-loop (TaqMan probe based) RT-PCR [229] and poly(A)-tailed (SYBR green based) RT-PCR [230], with improved specificity and sensitivity for miRNA expression analysis. Nonetheless, most published studies present conflicting data and have limitations in their cross-comparison of miRNA-expression profiles due to technical variations that include various reference genes being used to normalize the miRNA levels measured in body fluids and differences in blood collection (e.g., heparin contains an inhibitor of* Taq polymerase*) [231].

The optimal quantification of the target miRNAs involves data normalization using either stable reference genes under the study or accumulative values of the large scale miRNA-profiling data under study. However, as reference miRNAs (*RNU6B*,* 5S rRNA*) are reported to vary with the sample source and study type, former approach is suggested as preferred due to usage of global measure [232]. The addition of synthetic miRNAs from an unrelated organism such as* C. elegans* (cel-miR-39, cel-miR-54, and cel-miR-238) during miRNA isolation has also been proven useful for normalizing the data obtained by qRT-PCR [215]. Cheng et al. report plasma/serum volume as the best factor with which to standardize the amount of input miRNA [152]. However, studies are necessary for the identification of an accurate normalization protocol and empirical validation of stable endogenous control miRNAs for each type of body fluid.

For studying epigenetic modification, preliminary analysis involves methylated-DNA immunoprecipitation-chip (MeDIP-chip), validated differential methylation loci by bisulfate (BS) PCR and high throughput sequencing (BS-seq). The miRNA promoters in different cell types have been identified by genome-wide profiling of promoter associated chromatin marks through Dnase I hypersensitivity (DHS) mapping, chromatin immunoprecipitation (ChIP) followed by large-scale microarray analysis (DHS/ChIP-chip) or next-generation sequencing (DHS/ChIP-seq). DHS mapping identifies sites of open chromatin that are accessible to factors that influence gene expression. Active promoters are characterized by open chromatin regions enriched for both the H3K4me3 and H3K79me2 [42].

The use of miRNA as target for therapeutic tool has remained as challenge due to its redundancy that involves nonspecific targets. Antisense oligonucleotides primarily work as competitive inhibitors of miRNAs by binding to the mature miRNA strand and inducing degradation or stable duplex formation and making it unavailable for RISC formation. The microRNA-based therapeutic approaches have been recently summarized in several reviews [8, 197, 233]. To minimize the miRNA dysregulation, the low expressed miRNAs could be restored through molecular strategies such as mimic miRNA or adenovirus associated vectors (AAV) carrying miRNA encoding gene. Conversely, the upregulated miRNAs could be optimally managed through usage of antagomirs, locked nucleic acid (LNA) anti-miR, miRNA sponge, and miR-masks. However, the major problems complicating the use of* in vivo* miRNA therapeutics appear in the phase of tissue-specific delivery or the cellular uptake of sufficient amounts of synthetic oligo to achieve sustained target inhibition [233]. Application of antagomirs/anti-miRs in the animal model has shown few significant promising results [84, 130, 184, 188]. For example, Thum et al. demonstrated inhibition of interstitial fibrosis and attenuation of cardiac dysfunction by* in vivo* silencing of miR-21 through specific antagomir in a mouse pressure-overload-induced disease model with reduced cardiac ERK-MAP (extracellular signal-regulated kinase-mitogen-activated protein) kinase activity [184]. In another study, therapeutic inhibition of miR-208a by systemic subcutaneous delivery of LNA-modified oligonucleotide (anti-miR-208a) during hypertension-induced heart failure in rat model demonstrated the potent and sustained silencing of miR-208 in the heart with improved cardiac function and survival [188]. Besides, later on it was demonstrated that treatment with anti-miR-208a can result in significant increase of another miRNA, miR-19b (8.8-fold, *P* < 0.05) in the diseased group [185]. Therefore, more studies are warranted to analyze the impact of anti-miR treatment on the complex miRNA interaction network. Besides the therapeutic demonstration for such molecular approaches in animal models, challenges still remain for its application in humans. Towards this approach, human trials have been ongoing using locked nucleic acid approach and AAV owing to their efficiency and minimal toxicity related to off-target effects [233].

## 5. Conclusion

miRNAs play crucial role in immune system development, maintenance, and function. miRNA dysregulation is implicated in inflammatory pulmonary diseases and cardiac remodeling. Studies of miRNAs role in disease pathomechanisms have been undergoing translation to diagnostic area. In this context, two approaches to exploitation of miRNAs as biomarkers have been emerging: (1) characterization of the miRNA pattern typical for a given disease/condition (i.e., expression profiling) and (2) determination of miRNAs present in body fluids (circulating miRNA), which relative stability may add advantage to their potential use as biomarkers.

Regarding potential therapeutic applications of miRNAs, several approaches may be used to control pathological miRNA dysregulation. These range from the inhibition of pathologically upregulated miRNAs by anti-miRNA oligonucleotides/anatagomirs or miRNA mimics to potential delivery of a miRNA to maintain its physiological level in case of its downregulation in a disease. However, development of targeted therapies has remained challenging due to their possibilities of nonspecific targets or alteration in the gene-miRNAs and miRNA-miRNA interacting network. Additionally, the epigenetic modifications along with environmental factors as well as mutation within the seed region of miRNA are among the major issues affecting the miRNA based transcriptional control. Therefore, it would be imperative to evaluate the complex regulatory circuit between miRNA, mirSNPs, and epigenetic modifications that modulate the expression of numerous genes in the genome. Additional strategies, such as understanding of genes and the mechanism regulating the miRNAs, are still needed for early detection of disease progression for improving patient outcomes in lung and heart diseases. Taken together, measurement of altered miRNA expression serves as useful noninvasive approach for the diagnosis and prognosis of respiratory and cardiovascular disease. Further, the unique role of miRNAs should be explored for better clinical practices towards disease management.

## Figures and Tables

**Table 1:** Role of mirSNPs in lung inflammatory disease and cardiac remodeling events.

MicroRNA	SNP* risk variant (alleles)	Location	Putative role	References
Asthma				
miR-146a	rs2910164*C (C/G)	Pre-miRNA region of miR-146a	Protection/lower risk of asthma	[12, 13]
miR-149	rs2292832*T (C/T)	Pre-miRNA region of miR-149	Associated with lower risk of asthma	[12]
miR-152 family (miR-148a, -148b, and -152)	rs1063320*G (C/G)	*HLA-G *3′-UTR (at + 3142 position)	Protective against asthma only in children of asthmatic mothers	[11, 15]
Chronic obstructive pulmonary disease (COPD)				
miR-196a2	rs11614913*C (C/T)	Pre-miRNA region of miR-196a2	Target *Hoxb8*; decreased risk factor	[25]
miR-499	rs3746444*G (A/G)	Pre-miRNA regions of hsa-miR-499	Decreased risk factor	[25]
Cystic fibrosis (CF)				
miR-433 and -509-3p	rs10234329 (A/C)	*CFTR *3′-UTR	Mild CFTR mutation and may have a role in pathogenesis of CF	[33]
Pulmonary tuberculosis (PTB)				
miR-499	rs3746444*C (T/C)	Pre-miRNA regions of hsa-miR-499	Disease susceptibility varies among different populations	[25]
miR-146a	rs2910164*G (G/C)	Precursor miRNA sequence of miR-146a	Disease susceptibility varies among different populations; regulates cytokine signaling and TLR pathways	[25, 27]
Systemic lupus erythematosus (SLE)				
miR-569	rs1057233*T (T/C)	*SPI1 *3′-UTR	High susceptibility to SLE	[28]
miR-146a	rs57095329*G (G/A)	Promoter of miR-146a primary transcript	Associated with SLE	[29, 30, 34]
miR-146a	rs2431697*T (C/T)	Intergenic region between *PTTG1 *and miR-146a	Genetically associated with SLE in Europeans.	[31]
miR-3148	rs3853839*G (G/C)	*TLR7 *3′-UTR	Increased risk for SLE	[32]
Congenital heart disease				
miR-196a2	rs11614913*C (C/T)	Pre-miRNA region of miR-196a2	Increased risk	[24]
Hypertension (HT)				
miR-155	rs5186*C (A/C)	*AGTR1 *3′-UTR	Risk factor for HT and other CVDs	[35, 36]
miR-195 and -135	rs9818870 (C/T)	*MRAS *3′-UTR	Risk factor for HT and other CVDs	[37]
miR-31 and -584	rs7079 (C/A)	*AGT *3′-UTR	Risk factor for HT	[38]
Coronary heart disease				
miR-149	rs4846049*G (G/T)	*MTHFR *3′-UTR	Increased disease risk	[39]
Myocardial infarction				
miR-149 precursor	rs71428439*G (A/G)		Affects miR-149 maturation and regulates Puma protein in apoptosis	[40]
Dilated cardiomyopathy (DCM)				
miR-196a2	rs11614913*T (C/T)	Pre-miRNA region of miR-196a2	Increased disease risk	[41]
miR-499	rs3746444*G (A/G)	Pre-miRNA regions of hsa-miR-499	Increased disease risk	[41]

**Table 2:** Role of miRNAs in epigenetic regulation of lung inflammatory diseases and cardiac remodeling.

Disease and miRNA	Putative role in disease susceptibility	References
Idiopathic pulmonary fibrosis (IPF)		
miR-17~92 cluster	Hypermethylation and increased DNMT-1 expression: ↓miR-17~92 in lung biopsies and lung fibroblasts from IPF patients	[71]
SLE		
miR-21	Targets RASGRP1 and alters DNMT1 activity; DNA hypomethylation in disease state	[56]
miR-29b	Targets DNMT1; hypomethylation	[58]
miR-126	Inhibits DNMT1	[57]
miR-148a	Inhibits DNMT1	[56]
Myocardial infarction		
miR-21	Acetylation regulates miR-21 promoter in myocardial infarction	[72]

**Table 3:** miRNAs in inflammatory pulmonary diseases.

miRNA	Cell/tissue/body fluid (models)	miRNA regulation; validation method	Predicted target gene/possible effect	References
Asthma				
let-7 family (miR-98, let-7d, -7f, -7g, and -7i)	A549 cells and primary cultured T-cells (C)	Down; qRT-PCR, northern blotting	3′-UTR of IL-13	[81]
let-7 (a-e) and miR-200 (200b, and 141) families	Exosomes from BALF (H)	Differentially expressed; microarray, qRT-PCR	let-7 is associated with IL-13; miR-200 regulate EMT	[77]
miR-20b	Alveolar macrophages (M)	Down; qRT-PCR, transfection assay	⊥miR-20b: ↑VEGF	[82]
miR-21	Whole lung, macrophage, dendritic cells (M)	Up; microarray, qRT-PCR, ISH	IL-12p35	[74, 83]
miR-106a	Mouse macrophage (M), Jurkat (T-cell), Raji (B-cell), THP-1 cells (C)	Up; qRT-PCR, northern blotting	IL-10; ⊥mmu-miR-106a: ↑IL-10, ↓asthma features	[84, 85]
miR-126	Lower airway tissue (M)	Up; microarray, qRT-PCR	OBF.1	[75, 86]
miR-133a	Bronchial smooth muscle cells and bronchial tissues of mice (C + M)	Down; qRT-PCR	↓miR-133a: ↑RhoA	[76]
miR-145, -21, and let-7b	Lower airway tissue (M)	Up; qRT-PCR	↓miR-145: ↓T_H_2 cytokine (IL-13, IL-5, IFN-*γ*) production	[87]
miR-146a, -146b, and -28-5p	Human circulating T cells (H + C)	Down; microarray, qRT-PCR	Involved in T-cell activation	[67]
miR-146a, -146b, and -181a	Spleen CD4+ T lymphocytes (M)	Up; qRT-PCR	Th2 inflammation; proinflammatory factors in asthma	[88]
miR-155	Cell line and macrophages (C + M)	Up; microarray, qRT-PCR, transfection assay	⊥miR-20b; ↑Glucocorticoids	[89]
miR-221	Mice mast cell line (M + C), ASM cells cultured from bronchial biopsies (C + H)	Up; qRT-PCR, transfection assay	Augment cell proliferation and IL-6 production	[90, 91]
Chronic obstructive pulmonary disease (COPD)				
let-7c and miR-125b	Induced sputum (H)	Down; qRT-PCR	↓let-7c: ↑TNFR-II	[92]
miR-1 (muscle specific)	Muscle biopsy (H)	Down; qRT-PCR	IGF-1, HDAC4; ↓miR-1: ↓MRTF-SRF axis	[93]
miR-1, -499, -133, and -206	Plasma (H)	Up in patient with stable COPD; qRT-PCR	miR-499 with markers of inflammation NF-*κ*B p50	[94]
miR-7	Serum (H)	Up; qRT-PCR	—	[95]
mir-15/107 family (miR-15b, -424, and -107), -223, and -1274a	Lung tissue (H)	Up; microarray, qRT-PCR, ISH, and transfection assay	↑miR-15b: ↓SMAD7, SMAD7, decorin, and SMURF2	[96]
miR-18a and -365	Cell line, Lung tissue (C + H)	Up; microarray, qRT-PCR, ISH, and transfection assay	—	[96, 97]
miR-20a, -28-3p, -34c-5p, and -100	Serum (H)	Down; qRT-PCR	—	[95]
miR-26b, -29b, -101, -106b, -133b, -152, -483-5p, -532-5p, and -629	Plasma (H)	Down; TaqMan low-density array screening; qRT-PCR	miR-106b level negatively correlates with disease progression	[78]
miR-101 and -144	Human bronchial epithelial cell line (H + C), mice lung (C + M)	Up; qRT-PCR, luciferase assay, ISH	↑miR-101: ↓CFTR: ↑COPD	[98]
miR-146a	Primary lung fibroblasts (C + H)	Up; microarray, qRT-PCR	Degradation of COX-2 mRNA	[66]
miR-452	Alveolar macrophages (H)	Down; microarray and qRT-PCR	↑miR-452; ↓MMP12	[99]
miR-923 and -937	Lung tissue (H)	Down; microarray	Most downregulated in COPD	[96]
Cystic fibrosis (CF)				
miR-14 and -494	Several cell lines (C)	Up; qRT-PCR and luciferase assay	CFTR 3′-UTR	[100]
miR-101; -494	HEK293 cell line (C)	Up; luciferase assays	⊥CFTR	[101]
miR-101; -144	Human bronchial epithelial cell line, mice lung (C + M)	Up; qRT-PCR, luciferase assay, and ISH	↑miR-101: ↓*CFTR*: ↑COPD	[98]
miR-126	Bronchial epithelial cell line (C)	Down; qRT-PCR and luciferase assay	↓mir-126: ↑TOM1, TOLLIP	[102]
miR-138	Primary human airway epithelial cells (H + C)	Up; qRT-PCR and luciferase assay	Regulate CFTR	[103]
miR-145, -223, and -494	*In vivo * bronchial brushings and cell lines (H + C)	Up; qRT-PCR and luciferase assay	Regulate CFTR	[104]
miR-145 and -494	Nasal epithelial tissues (H)	Up; qRT-PCR	↑miR-145: ↓*SAMD3*; ↑miR-494: ↓CFTR	[105]
miR-155	Cell line, mouse (C + M)	Up; microarray and qRT-PCR	FGF7/KGF; ↑miR-155: ↓SHIP1, ↑IL-8	[106–109]
miR-215	Ex vivo CF lung epithelial cells (C)	Up; qRT-PCR	↑IL-8	[108]
miR-384, -494, and -1246	Airway epithelial cells (C)	Down; qRT-PCR and luciferase assay	3′- UTR of SLC12A2; ⊥CFTR	[100]
miR-509-3p and -494	Primary human airway epithelial cells (H + C)	Up; qRT-PCR	Regulate CFTR expression	[110]
Idiopathic pulmonary fibrosis (IPF)				
let-7d, miR-26, and miR-30 family	Lung biopsies and lung epithelial cells (M + C)	Down; microarray, qRT-PCR, and luciferase assay	↓let-7d: ↑HMGA2	[111]
miR-17~92 cluster	Lung biopsies (H)	Down; microarray and qRT-PCR	Metalloproteinases, collagen, and TGF	[112, 113]
miR-21	Lung biopsies and serum (M + H)	Up; microarray, qRT-PCR, ISH, and luciferase assay	↑TGF-*β*, ⊥SMAD7; enhance EMT and IPF	[62–64, 114]
miR-29	Lung tissues and pulmonary fibroblasts (M + C)	Down; microarray, qRT-PCR, ISH, and luciferase assay	Integrin, alpha 11; ADAMTS9; ADAM12, and nidogen-1	[115–117]
miR-34a	Lung (M)	Up; microarray, qRT-PCR, ISH, and luciferase assay	—	[63]
miR-154, -134, -299–5p, -410, -382, -409–3p, -487b, -31, and -127	Lung tissue + primary normal human lung fibroblast cells (H + C)	Up; microarray and qRT-PCR	SMAD3 to promoter of miR-154	[118]
miR-200 family (a-c)	Lung (M)	Down	Inhibit *TGF- β1* induced EMT of alveolar epithelial cells	[61]
Acute lung Injury (ALI) and inflammation				
miR-127	Mouse macrophage cell line (M + C)	Down; luciferase assay and microarray	IgG Fc*γ*RI (CD64); ↑miR-127: ↓cytokine from macrophages	[119]
Let-7; miR-21; -146; -155	Lung (M)	Up; TaqMan low density arrays and qRT-PCR	Let-7 regulates IL-6; miR-146 –SMAD-4; miR-155 – SOCS-1	[120]
miR-32*; -466-5p; -466-3p	Rat alveolar epithelial cell (R)	Up; microarray and qRT-PCR	—	[121]
miR-146a	Lung, macrophage cell line (R + C)	Up	↑miR-146a: ⊥TNF-*α*, IL-6, IL-1*β*, IRAK-1, TRAF-6;	[122, 123]
miR-181b	Cell line (C)	Up; microarray and qRT-PCR	↑miR-181b: ⊥Importin-*α*3; regulate NF-*κ*B signaling	[124]
Pulmonary arterial hypertension (PAH)				
miR-17/92 cluster (miR-17-5p, -20a)	hPAECs (H + C)	Up; qRT-PCR; reporter gene assay	↑miR-17/92; ↓BMPR2 protein; ↑STAT3	[125]
miR-21	hPASMC (H + C)	Up; qRT-PCR and western blotting	↑miR-21: ↓(*PDC4*, *SPRY2*, PPAR*α*, BMPR2); role in cell proliferation and migration	[126, 127]
miR-23b, -130a, and -191	Blood buffy coat (H)	Up; qRT-PCR	miR-23b: ⊥BMPR1b; *miR-130a*:⊥BMPR1b, SMAD2	[128]
miR-124	hPASMC (H + C)	Down; qRT-PCR	NFATc1, CAMTA1 and PTBP1; inhibitor of NFAT signaling	[129]
miR-145	hPASMC (H + C) and mouse (M)	Up; qRT-PCR	↑miR-145; BMPR2 mutations; vessel remodeling	[130]
miR-204	hPASMC (H + C), mouse (M), and rat lung (R)	Down; qRT-PCR	↑STAT3, ↑SHP2;accounts for the proliferative and antiapoptotic phenotypes	[131]
miR-206	Lung tissue + hPASMC (H + C) and mouse (M)	Down; qRT-PCR	↓*Notch-3*, ↑HIF-1*α*/Fhl-1 pathway; regulate proliferation	[132, 133]
miR-424 and 503	hPAECs (H + C), mouse (M) and rat (R) lung endothelial cell (C)	Down; qRT-PCR	↑FGFR1, FGF2; antiproliferative effects	[134]
miR-451 and -1246	Blood (H)	Down; qRT-PCR	miR-206: Titin; miR-1246: *caveolin1*, *GSK*3*β*,* and cadherin2 *	[128]

↑: increased level; ↓: decreased level; ⊥: inhibition. Investigating models: mouse (M), rat (R), cell culture (C), and human (H). ADAM metallopeptidase with thrombospondin type 1 motif, 9 (ADAMTS9); bone morphogenetic protein receptor type Ib (BMPR1b); epithelial-mesenchymal transition (EMT); fibroblast growth factor 2 (FGF2); fibroblast growth factor receptor 1 (FGFR1); human pulmonary arterial endothelial cells (HPAECs); in situ hybridization (ISH); insulin-like growth factor 1 (IGF-1); interleukin-1 receptor-associated kinase 1 (IRAK1); interleukin-8 (IL-8); histone deacetylase 4 (HDAC4); matrix metalloproteinase-12 (MMP-12); nuclear factor-*κ*-B (NF*κ*-B); nuclear factor of activated T cells (NFAT); peroxisome proliferator-activated receptor-*α* (PPAR*α*), POU domain class 2 associating factor 1, also named Oct binding factor 1 (OBF.1); programmed cell death protein 4 (PDC4); pulmonary artery endothelial cells (PAECs); human pulmonary artery smooth muscle cells (hPASMCs); human pulmonary arterial endothelial cells (HPAECs), quantitative real-time PCR (qRT-PCR); signal transducer and activator of transcription 3 (STAT3); solute carrier family 12 (sodium/potassium/chloride transporters), member 2 (SLC12A2); sprouty 2 (SPRY2); suppressor of cytokine signaling proteins (SOCS); target of Myb1 (TOM1); Toll-interacting protein (TOLLIP); TNF receptor associated factors (TRAFs); transforming growth factor beta (TGF-*β*); tumor necrosis factor receptor type II (TNFR-II); vascular endothelial growth factor (VEGF).

**Table 4:** miRNAs in cardiovascular diseases.

miRNA	Cell/tissue/body fluid (models)	Regulation	Validation method	Predicted target gene/possible effect	References
Atherosclerosis					
miR-21	Plaques and arteries (H)	Up	qRT-PCR	Signal transduction; regulation of transcription; vesicular transport	[142, 143]
miR-26, -30, and -125a	Plaque tissue (H)	Up	qRT-PCR	Signal transduction; regulation of transcription; vesicular transport	[144]
miR-34a	Plaques and arteries (H)	Up	qRT-PCR	Signal transduction; regulation of transcription; vesicular transport	[143]
miR-146a	Plaques and arteries (H)	Up	qRT-PCR	Signal transduction; regulation of transcription; vesicular transport	[142, 143, 145]
miR-146b-5p	Plaques and arteries (H)	Up	qRT-PCR	Signal transduction; regulation of transcription; vesicular transport	[143]
miR-181b	Aortic intima (M) and plasma (H)	Down	qRT-PCR	Importin-*α*3; ⊥NF-*κ*B signalling and atherosclerotic lesion formation	[146]
miR-210	Plaques and arteries (H)	Up	qRT-PCR	Potential biomarker	[143]
miR-712	Mouse arterial endothelial cell (M + C)	Up	qRT-PCR	↓TIMP3; ↑MMPs and ADAMs	[147]
Acute myocardial infarction (AMI)					
Let-7b	Plasma (H)	Up	qRT-PCR	Differentiate AMI patients from healthy controls	[148]
miR-1	Plasma (H), serum (M), and rat cardiac cells (R + C)	Up	qRT-PCR	Potential biomarker for AMI	[149–154]
miR-21	Heart (R)	Down	qRT-PCR	Expression signature in early phase of AMI	[155]
miR-30a and -195	Plasma (H)	Down	qRT-PCR	Differentiate AMI patients from healthy controls	[148]
miR-122	Plasma (H)	Down	qRT-PCR	Potential biomarker for AMI	[151, 156]
miR-133a and -b	Plasma and whole blood (H)	Up	qRT-PCR	Potential biomarker for AMI	[150, 151, 154, 156]
miR-133 and -328	Plasma and whole blood (H)	Up	qRT-PCR	Potential biomarker for AMI	[157]
miR-126	Plasma (H)	Down	qRT-PCR	Potential biomarker for AMI	[153]
miR-155	Monocytes, B-cells, T-cells (H), and knockout mice (M)	Up	miRNA profiling and qRT-PCR	*TLR*- pathway, NF-*κ*B, FADD, *Rip-1*; proinflammatory miRNA	[158]
miR-208a and -499	Plasma (H)	Up	qRT-PCR	Potential biomarker for AMI	[154, 159, 160]
Cardiac hypertrophy					
miR-1	Heart and skeletal muscle (M + R) and coronary artery cells (H)	Down	Nothern blot; qRT-PCR	Twinfilin-2	[161]
miR-21	Heart (M)	Up	Northern blot and qRT-PCR	—	[162]
miR-22	Cardiac and muscle specific (M)	Up	qRT-PCR and western blot	Sirt1 and HDACY	[163]
miR-27b	Cardiomyocytes (M)	Up	Western blot and qRT-PCR	PPAR-*γ*	[164]
miR-29a	Plasma (H)	Up	qRT-PCR		[165]
miR-30	Heart (R), cardiac tissue, and plasma (H)	Down	qRT-PCR	*Beclin-1*; ↑Ang II: ↓miR-30a: ↑*Beclin-1*: excessive autophagy: myocardial hypertrophy	[166]
miR-133	Human (H), mouse (M), and rat (R) heart	Down	qRT-PCR and northern and western blot	RhoA, Cdc42, Nelf-A/WHSC2	[167, 168]
miR-155	Leukocytes (M)	Up	—	Promotes cardiac inflammation, hypertrophy, and failure	[169]
miR-214	Heart and cardiomyocytes (R)	Up	Luciferase assay and western blot	↑miR-214; ↓EZH2	[136]
Cardiac fibrosis					
miR-21	Cardiac fibroblast (H)	Up	—	SPRY1	[170]
miR-21	Cardiac myocytes (M)	Up	qRT-PCR	⊥*PTEN*; protects against cardiac ischemia/reperfusion Injury	[171, 172]
miR-29	Cells, mouse (M), and human (H) cardiac tissue	Up	miRNA microarray and qRT-PCR	Inflammatory cytokines	[173]
miR-133	Heart (M)	Down	qRT-PCR	Col1A1	[170, 174]
miR-26a	Heart (M)	Down	qRT-PCR	CTGF and Col1A1	[175]
miR-30	Heart (H + R), cardiac fibroblast, and myocytes (R + C)	Up	qRT-PCR	↑miR-30: ↓*CTGF *	[176]
miR-122	Endomyocardial biopsies (H)	Down	qRT-PCR	⊥TGF-*β*1; progression of myocardial fibrosis in aortic stenosis	[177]
Coronary artery disease (CAD)					
miR-17/92a cluster, -126, -145, and -155	Plasma (H)	Down	qRT-PCR	Potential biomarker for CAD	[178]
miR-21	Endothelial cells and vessel wall (H)	Up	qRT-PCR	Potential biomarker for CAD	[179]
miR-106b/25 cluster, -17/92a cluster, -21/590-5p family, -126*, and -451	Plasma (H)	Up	qRT-PCR	May affect inflammation, hypoxia, angiogenesis, apoptosis, and extracellular matrix (ECM) degradation	[180]
miR-126	Endothelial cells and vessel wall (H)	Down	qRT-PCR	Potential biomarker for CAD	[151, 179]
miR-133a	Endothelial cells and vessel wall (H)	Up	qRT-PCR	Potential biomarker for CAD	[179]
miR-135a	PBMC (H)	Up	qRT-PCR	Potential biomarker for CAD	[181]
miR-147	PBMC (H)	Down	qRT-PCR	Potential biomarker for CAD	[181]
miR-155	Endothelial cells and vessel wall (H)	Down	qRT-PCR	Potential biomarker for CAD	[151, 179]
miR-208a	Endothelial cells and vessel wall (H)	Up	qRT-PCR	Potential biomarker for CAD	[151]
miR-221	Endothelial cells and vessel wall (H)	Down	qRT-PCR	Potential biomarker for CAD	[179]
miR-370	Endothelial cells and vessel wall (H)	Up	qRT-PCR	Potential biomarker for CAD	[179]
Heart failure					
miR-1	Muscle (H)	Down	qRT-PCR	*GATA 14 *and* MEF2A *	[182–184]
miR-16, -20b, -93, -106b, -223, and -423-5p	Plasma (R)	Up	qRT-PCR	Expression level changes during progression of hypertension-induced heart disease	[185]
miR-21	Plasma, muscle, and heart tissue (H)	Up	qRT-PCR	*PTEN *	[182–184]
miR-23a	Plasma, muscle, and heart tissue (H)	Up	qRT-PCR	*MuRF1 *	[182–184]
miR-29b	Plasma, muscle, and heart tissue (H)	Down	qRT-PCR	*Col1A1, Col1A2, *and* Col3A1 *	[182–184]
miR-30	Plasma, muscle, and heart tissue (H)	Down	qRT-PCR	*CTGF *	[176, 182–184]
miR-125	Plasma, muscle, and heart tissue (H)	Up	qRT-PCR	—	[182–184]
miR-126	Plasma (H)	Down	qRT-PCR	Useful biomarker for heart failure	[186]
miR-133	Right atrial appendages (H)	Down	qRT-PCR	*CTFG* and *SRF*	[187]
miR-208a	Cardiac tissue and plasma (M + R)		Microarray profiling; qRT-PCR		[188]
miR-210	Plasma (M + H), H9c2 (C), and mononuclear cells (M + H)	Up	qRT-PCR	Repress ISCU, leading to repression of mitochondrial respiration	[189]
miR-423-5p; -320, -22, and -92b	Plasma (H) and serum (H)	Up	qRT-PCR	Positive correlation of miR-423-5p with BNP and NT-proBNP	[190–192]

↑: increased level; ↓: decreased level; ⊥: inhibition. Investigating models: mouse (M), rat (R), cell culture (C), and human (H).A disintegrin and metalloproteases (ADAMs); collagen, type I, alpha 2 (Col1A2); collagen, type III, alpha 1 (Col3A1); connective tissue growth factor (CTGF); cytoplasmic SH2 domain containing protein tyrosine phosphatase (SHP2); enhancer of zeste homolog 2 (EZH2); Fas-associated death domain (FADD); iron-sulfur cluster assembly protein (ISCU); matrix metalloproteinases (MMPs); myocyte enhancer factor 2A (MEF2A); muscle ring-finger protein-1 (MuRF1); nuclear factor-kB (NF-kB); phosphatase and tensin homolog (PTEN); receptor-interacting protein 1 (RIP1); serum response factor (SRF); sirtuin 1 (Sirt1); proliferator-activated-receptor-*γ* (PPAR-*γ*); sprouty homolog 1 (SPRY1); Toll-like receptor (TLR); tissue inhibitor of metalloproteinase 3 (TIMP3).
